# Supplementary material for: The iron-dependent repressor YtgR is a tryptophan-dependent attenuator of the trpRBA operon in Chlamydia trachomatis
Source: Nat Commun. 2020 Dec 22;11:6430. doi: 10.1038/s41467-020-20181-5 (PMC7755916; doi:10.1038/s41467-020-20181-5)
Supplement: Supplementary file 1 — Supplementary Information [file 41467_2020_20181_MOESM1_ESM.pdf]

Supplementary Information

Pokorzynski, N. D., Hatch, N. D., Ouellette, S. P. & Carabeo, R. A. The iron-dependent repressor YtgR is a tryptophan-dependent attenuator of the *trpRBA* operon in *Chlamydia trachomatis*. *Nat. Commun.* (2020).

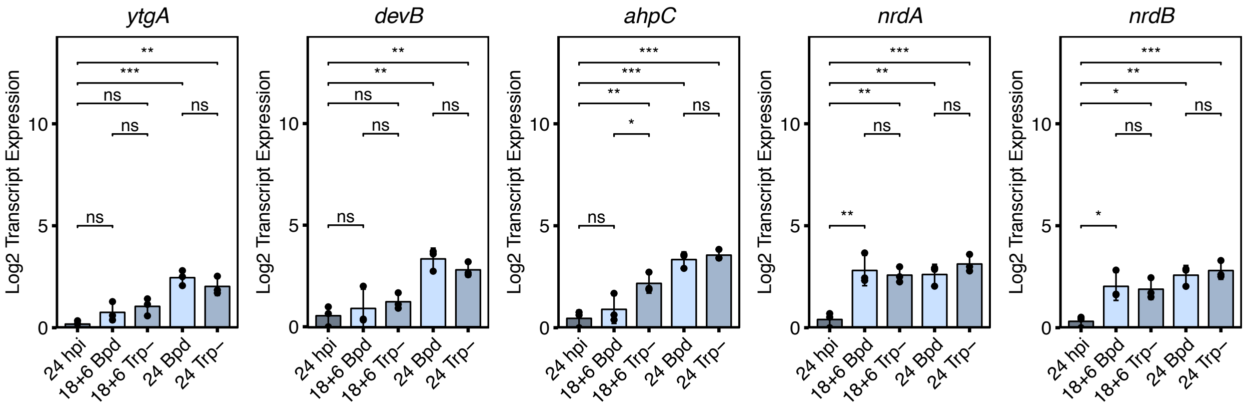

Supplementary Fig. 1 Previously characterized iron-regulated genes converge at common expression endpoint in response to both iron-limited and tryptophan-starved persistence. Transcript expression of genes previously identified as iron-regulated in *C. trachomatis* serovar L2. RNA was isolated from infected HeLa cells (MOI 2) and transcript abundance was quantified by RT-qPCR after normalization to genome copy number. Note that *ytgA* is a known regulatory target of YtgR. Data were collected from three independent experiments (N=3) and are plotted as mean +/- s.d. Statistical significance in all panels was determined by one-way ANOVA followed by Tukey's post-hoc test of honest significant differences (two-tailed). * = $p < 0.05$, ** = $p < 0.01$, *** = $p < 0.001$.

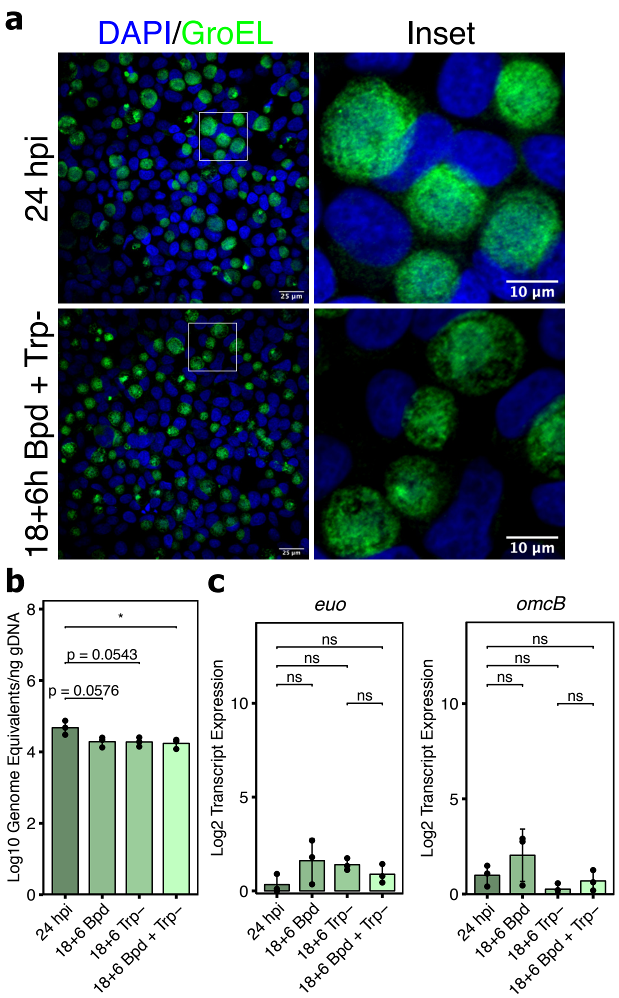

Supplementary Fig. 2 Combined stress of iron and tryptophan limitation does not further induce persistence in *C. trachomatis*. **a** Representative immunofluorescent confocal micrographs of *C. trachomatis* L2-infected HeLa cells indicating inclusion morphology following the combination of iron and tryptophan limitation for 6h. Note that inclusions are very similar to those observed following 6h tryptophan limitation in Fig. 1a. N=3. **b** *C. trachomatis* L2 genome copy number quantified as in Fig. 1b for the combined stress condition. **c** Transcript expression of persistence biomarkers *euo* and *omcB* following combined stress treatment. Data in plots were collected from three independent experiments (N=3) and represent the mean +/- s.d. Statistical significance was determined by one-way ANOVA followed by Tukey's post-hoc test of honest significant differences (two-tailed). * = $p < 0.05$, ** = $p < 0.01$, *** = $p < 0.001$.

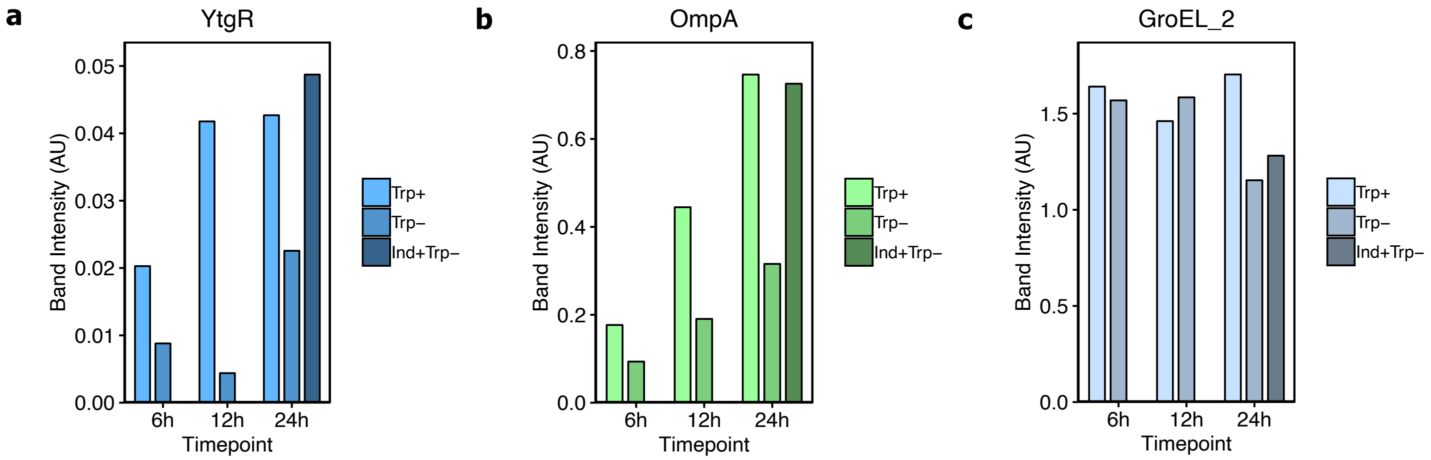

Supplementary Fig. 3 Quantification of protein expression levels of **a** YtgR, **b** OmpA and **c** GroEL_2 following media-defined tryptophan limitation as displayed in Figure 2b (N=1). Densitometric quantification of YtgR, OmpA and GroEL_2 levels were normalized to the intensity of the GroEL_1 band. Note that GroEL_1 contains zero tryptophan codons.

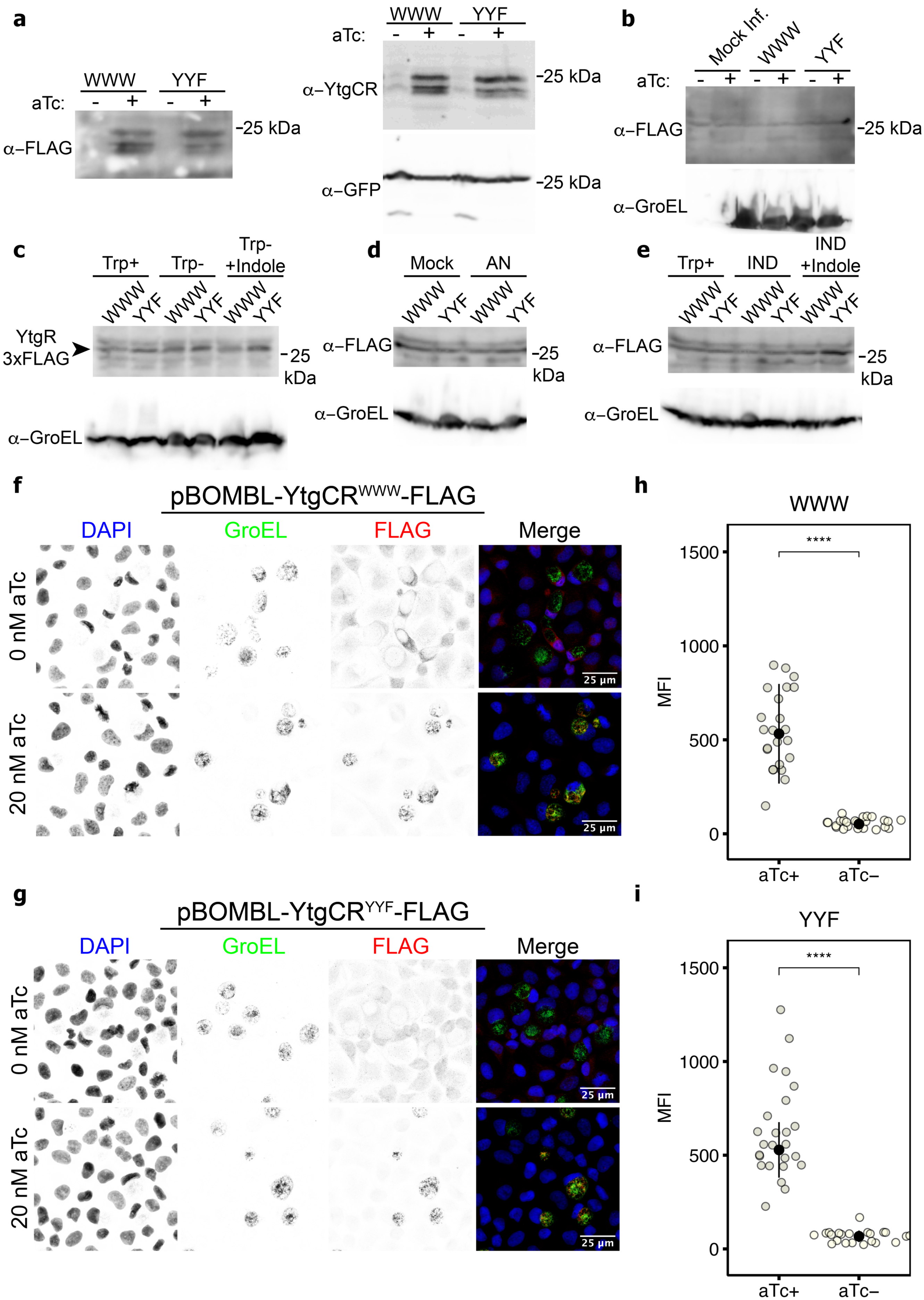
a
WWW
YYF
aTc:
- + - +
α–FLAG
-25 kDa
α–YtgCR
α–GFP
b
Mock Inf.
α–GroEL
c
Trp+
Trp-
Trp-
+Indole
YtgR
3xFLAG
-25
kDa
d
Mock
AN
e
IND
IND
+Indole
f
pBOMBL-YtgCR^{WWW}-FLAG
DAPI
GroEL
FLAG
Merge
0 nM aTc
20 nM aTc
25 µm
g
pBOMBL-YtgCR^{YYF}-FLAG
h
WWW
MFI
1500
1000
500
0
****
aTc+
aTc–
i
YYF

Supplementary Fig. 4 *C. trachomatis* L2 pBOMBL-YtgCR-FLAG transformants specifically express YtgCR-FLAG in the presence of anhydrotetracycline. **a** Representative immunoblot of pBOMBL-YtgCR-FLAG constructs expressed in *E. coli*. Left blot shows detection of FLAG epitope following 3h with (+) or without (-) induction by 100 nM anhydrotetracycline (aTc) whereas the right blots show detection of YtgR cleavage products from the same lysates with the constitutively expressed plasmid-borne GFP as a loading control. Images are representative of three independent experiments (N=3). **b** Representative immunblot of FLAG epitope from lysates collected from uninfected or pBOMBL-YtgCR-FLAG infected HeLa cell lysates following 6h of 200 nM aTc induction starting at 18 hpi. Detection of chlamydial GroEL was used as a control for chlamydial protein content and a marker for infection. At least two other derivatives of this experiment produced the same result (N=3). **c** Representative immunoblot of FLAG epitope from lysates collected from HeLa cells infected with *C. trachomatis* strains transformed with the WWW or YYF variant of pBOMBL-YtgCR-3xFLAG under tryptophan-replete (Trp+), tryptophan-deplete (Trp-) or indole-supplemented tryptophan-deplete media (Trp- +Indole). Treatment began at 8 hpi and 200 nM aTc was added at 16 hpi prior to lysate collection at 24 hpi. At least one other derivative of this experiment produced the same result (N=2). **d** Same as in **c** with the exception that infected cells were treated with 1.0 µg/mL of the non-competitive leucyl-tRNA synthetase inhibitor AN3365 at the time of aTc induction (16 hpi). Image is representative of three independent experiments (N=3). **e** Same as in **d** with the exception that infected cells were treated at 16 hpi with 240 µM of the competitive tryptophanyl-tRNA synthetase inhibitor indolmycin in tryptophan-deplete media (IND) or in indole-supplemented tryptophan-deplete media (IND + Indole). At least two other derivatives of this experiment produced the same result (N=3). **f** Representative immunofluorescent confocal micrographs of McCoy B mouse fibroblasts infected with the WWW (wild-type) variant of the pBOMBL-YtgCR-FLAG construct (MOI 5) and treated with 20 nM aTc for 6h to induce YtgCR-FLAG expression. Monoclonal mouse antibody against the GroEL_1 – GroEL_3 antigens was used a counterstain for *C. trachomatis*. **g** Same as in **f** for the YYF variant of the transformant. **h** Quantification of FLAG-594 signal from aTc-induced (aTc+) and -uninduced (aTc-) YtgCR^{WWW}-FLAG transformants. Induction was performed in tryptophan-replete media as described for experiments in Figure 2c-f. **i** Same as in **h** for YtgCR^{YYF}-FLAG transformants. Black dot in dotplots indicates the population median and the error bars represent the median absolute deviation. Data in plots represent one field from two independent biological replicates without random sampling (N=2). Statistical significance in all panels was determined by pairwise two-sided Wilcoxon rank-sum test. * = $p < 0.05$, ** = $p < 0.01$, *** = $p < 0.001$.

a

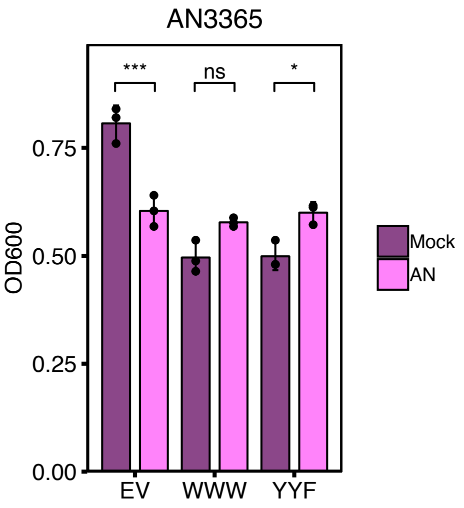

b

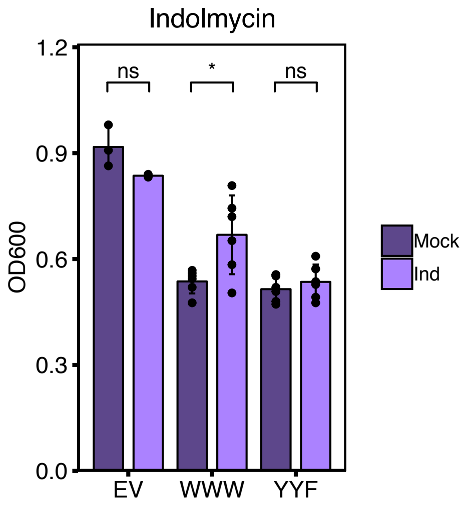

Supplementary Fig. 5 Inhibition of YtgCR-3xFLAG expression by addition of AN3365 or indolmycin rescues *E. coli* growth. **a** Optical density measured at 600 nm (OD_{600}) from BL21 (DE3) *E. coli* cultures transformed with empty vector pET151 (EV), pET151-YtgCR^{WWW}-3xFLAG (WWW) or pET151-YtgCR^{YYF}-3xFLAG (YYF) following treatment with either 10 µg/mL AN3365 (AN) or mock treatment. OD_{600} was measured at the end of each experiment. Note that AN3365 treatment alone significantly inhibited *E. coli* growth and that inhibition of YtgCR expression rescued growth back to EV levels in the presence of AN3365. Data were collected from three independent experiments (N=3). **b** Same as in **a** for indolmycin (Ind)-treated experiments. Note here that indolmycin treatment alone does not affect *E. coli* growth. Data were collected from three independent experiments for EV (N=3) and six independent experiments for WWW and YYF (N=6). Statistical significance in all panels was determined by a Two-way factorial ANOVA followed by Tukey's post-hoc test for honestly significant differences (two-tailed). * = $p < 0.05$, ** = $p < 0.01$, *** = $p < 0.001$.

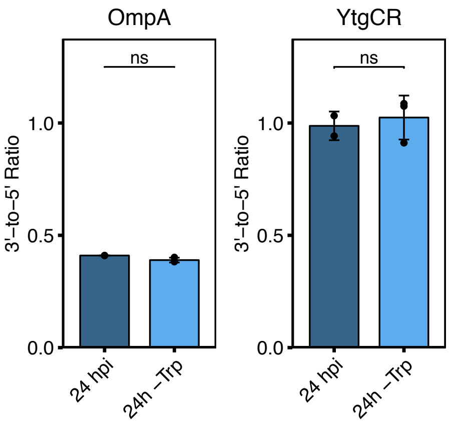

Supplementary Fig. 6 Media-defined tryptophan limitation does not induce 3’ transcriptional polarity of YtgCR. Analysis of 3’ transcriptional polarity determined by RT-qPCR. *C. trachomatis* L2-infected HeLa cells (MOI 2) were tryptophan starved by media replacement for 24h at the start of infection. YtgCR transcriptional polarity was measured as in Fig. 4b and 4c. OmpA transcriptional polarity was included as a negative control since it did not polarize in response to 14h indolmycin treatment (Fig. 4b). Data were collected from at least two independent experiments (N=2-3) and are plotted as mean +/- s.d. Statistical significance in all panels was determined by Welch’s pairwise two-sided unpaired *t*-test for unequal variance. * = $p < 0.05$, ** = $p < 0.01$, *** = $p < 0.001$.

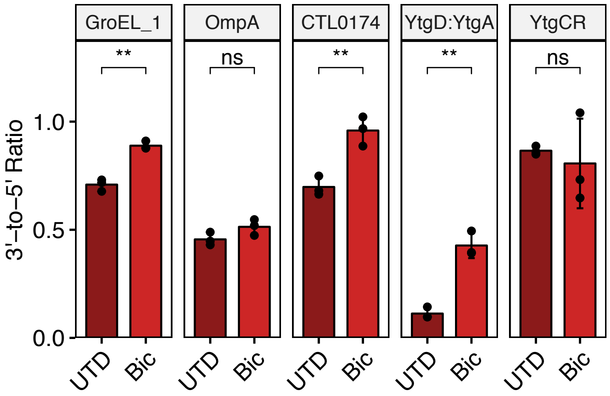

Supplementary Fig. 7 3' transcriptional polarity of YtgCR is not regulated by the transcription termination factor Rho. Analysis of 3' transcriptional polarity determined by RT-qPCR for genes assayed in Fig. 4b. *C. trachomatis* L2-infected HeLa cells (MOI 2) were untreated (UTD) or treated with 25 µg/mL of the Rho inhibitor bicyclomycin (Bic) for 6h to assess Rho-dependence. Transcriptional polarity of the *ytg* operon (YtgD:YtgA) was included as a positive control for Rho-dependent regulation of transcription termination. Data were collected from three independent experiments (N=3) and are plotted as mean +/- s.d. Statistical significance in all panels was determined by Welch's pairwise two-sided unpaired *t*-test for unequal variance. * = $p < 0.05$, ** = $p < 0.01$, *** = $p < 0.001$.

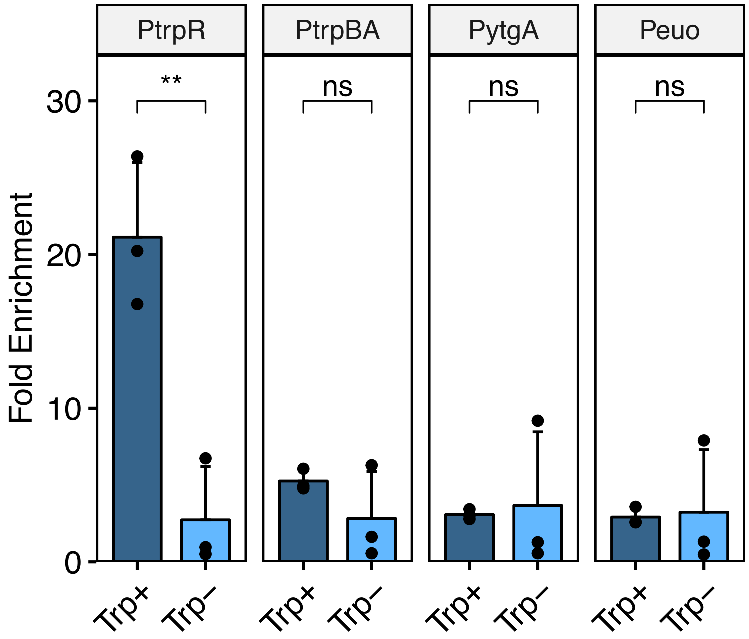

Supplementary Fig. 8 Chromatin immunoprecipitation (ChIP)-qPCR of TrpR-bound promoter fragments in the presence or absence of tryptophan. TrpR was immunoprecipitated using a polyclonal antibody raised against a peptide fragment of *C. trachomatis* L2 TrpR. Tryptophan depletion was carried out for 12h starting at 12 hpi. The promoters for *ytgA* and *euo* were included as negative controls. Data were collected from three independent experiments (N=3) and are plotted as mean +/- s.d. Statistical significance in all panels was determined by Welch's pairwise two-sided unpaired *t*-test for unequal variance. * = $p < 0.05$, ** = $p < 0.01$, *** = $p < 0.001$.

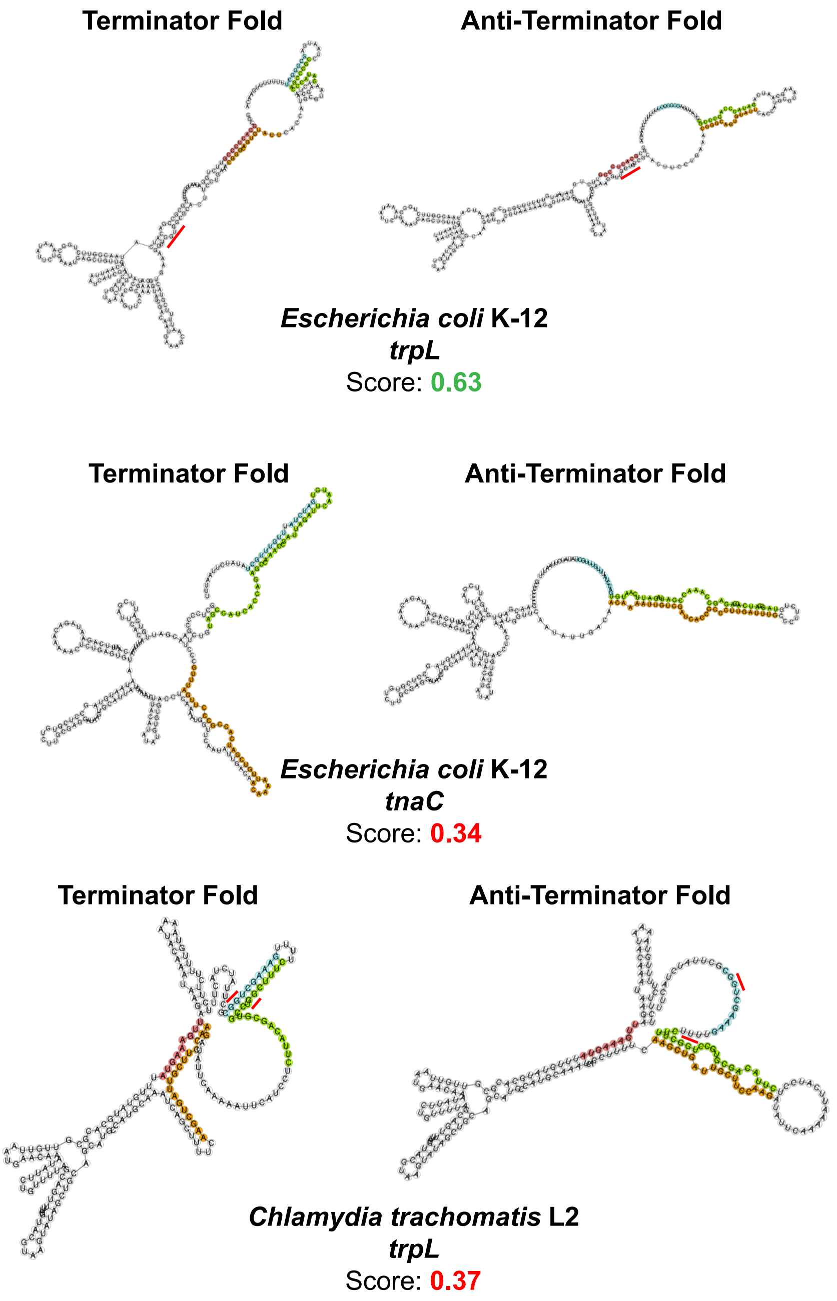

Supplementary Fig. 9 The *trpRBA* intergenic region is not predicted to form a *cis*-attenuator termination structure. The PASIFIC algorithm was used to predict attenuator function from three regions: the intergenic region upstream of the *E. coli trpEDCBA* operon containing the *trpL* leader peptide, the intergenic upstream of the *E. coli tnaAB* operon containing the *tnaC* leader peptide and the intergenic region upstream of the *C. trachomatis trpBA* ORFs containing the putative *trpL* leader peptide. *E. coli trpL* was used a positive control for attenuator function, whereas the *tnaC* leader sequence was used as a negative control as it does not rely on a terminator/anti-terminator formation for its regulatory function. The PASIFIC algorithm predicts regions of base pairing that could form putative terminators (blue and green base-pairing) and anti-terminators (green and orange base-pairing) and computes a score for the prediction. A score of 0.50 or higher is considered reliable. Whereas the *E. coli trpL* leader sequence was identified as a strong positive attenuator sequence and reproduces base-pairing predicted in its original description, the *tnaC* leader sequence is not predicted to form a terminator/anti-terminator structure. Likewise, the intergenic region of the *C. trachomatis trpRBA* operon is not predicted to form a functional terminator/anti-terminator structure. Notably, as the red lines indicate, the tryptophan codons in the putative *C. trachomatis trpL* are predicted to be involved in the base pairing interaction of the terminator hairpin, precluding ribosomes from "sensing" these codons to form the terminator hairpin. This is consistent with the prediction of poor attenuator function. Note that this is not the case for the *E. coli trpL* leader sequence. Additionally, PASIFIC predicts the transcript termination site when one is not known. For this analysis, all predictions were determined using an unknown termination site. For *C. trachomatis*, termination is predicted to occur prior to the end of the putative *trpL* peptide coding sequence, suggesting that this region is possibly misannotated. See Methods for complete description of analysis.

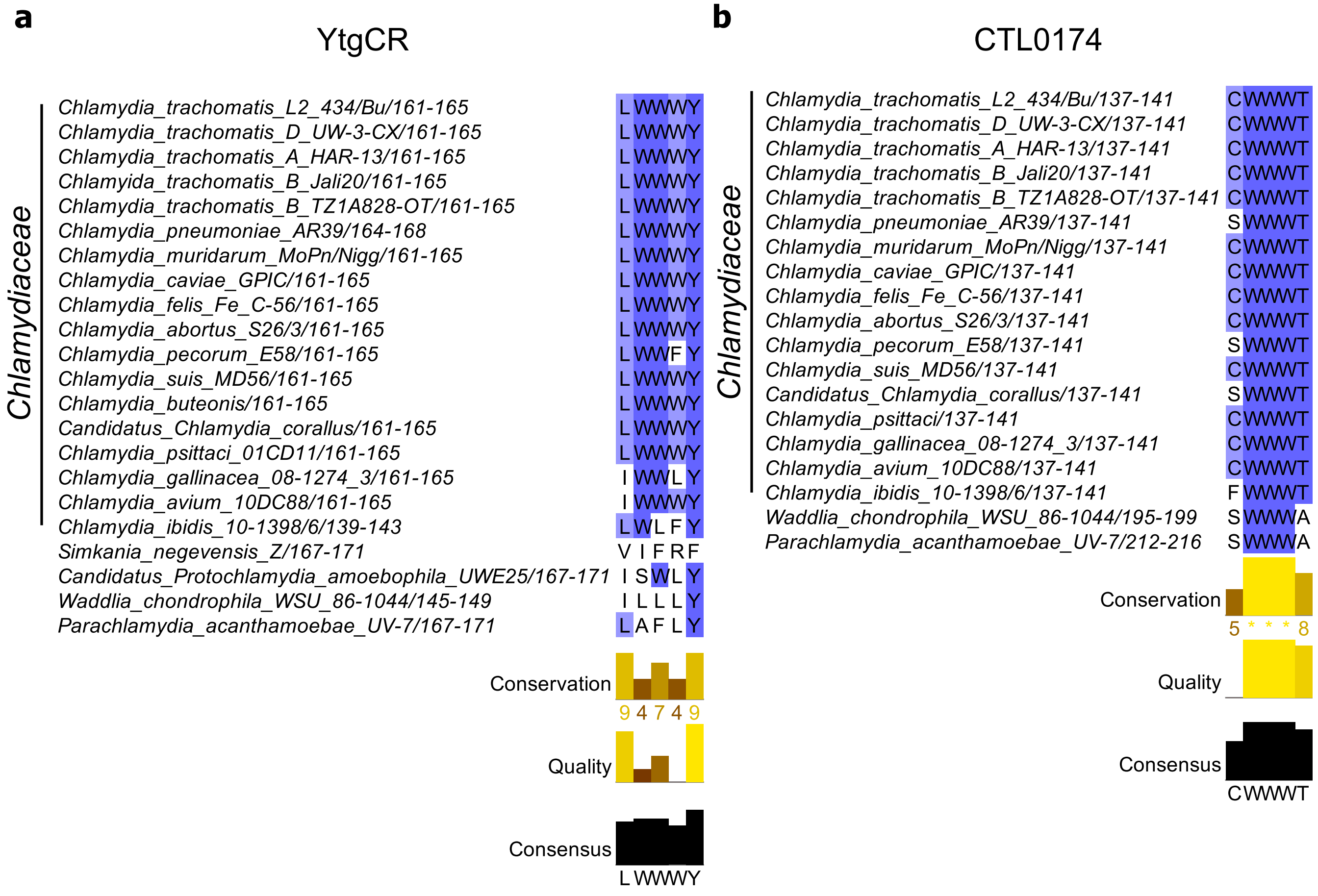

Supplementary Fig. 10 Sequence analysis of WWW motifs across the *Chlamydiales* order. **a** MUSCLE Multiple sequence alignment (MSA) of CTL0325 (YtgCR) across the *Chlamydiales*. Note that the WWW motif is well conserved across the *Chlamydiaceae* but poorly conserved in the environmental chlamydiae. **b** Same as in **a** for CTL0174. Note that the WWW motif of CTL0174 is perfectly conserved even in the homolog of environmental chlamydiae. The coloration of the residues indicates the conservation across the MSA, with darker shades indicating higher conservation. Differences in the species included in each analysis reflects presence of the homolog.

Supplementary Table 1. Plasmids used in this study

Plasmid	Relevant Details	Application	Figure
pET151-EV	Circularized empty vector	Expression of YtgCR in *E. coli*	Fig. 3
pET151-YtgCR(WWW)-3xFLAG	Expression of YtgCR with intact WWW motif and C-terminal 3xFLAG epitope w/ N-terminal 6xHis and V5 tags	Expression of YtgCR in *E. coli*	Fig. 3
pET151-YtgCR(YYF)-3xFLAG	Expression of YtgCR with WWW motif mutated to YYF and C-terminal 3xFLAG epitope w/ N-terminal 6xHis and V5 tags	Expression of YtgCR in *E. coli*	Fig. 3
pBOMBL-YtgCR(WWW)-FLAG	Expression of YtgCR in *C. trachomatis* with intact WWW motif and C-terminal FLAG epitope	Expression of YtgCR in *C. trachomatis*	Fig. 2C-D, Fig. S3
pBOMBL-YtgCR(YYF)-FLAG	Expression of YtgCR in *C. trachomatis* with WWW motif mutated to YYF and C-terminal FLAG epitope	Expression of YtgCR in *C. trachomatis*	Fig. 2C-D, Fig. S3
pRACE	Sequencing vector for RACE products	RACE Sequencing	Fig. 5B-C
