# Supplementary material for: The iron-dependent repressor YtgR is a tryptophan-dependent attenuator of the trpRBA operon in Chlamydia trachomatis
Source: Nat Commun. 2020 Dec 22;11:6430. doi: 10.1038/s41467-020-20181-5 (PMC7755916; doi:10.1038/s41467-020-20181-5)
Supplement: Supplementary file 3 — Description of Additional Supplementary Files [file 41467_2020_20181_MOESM3_ESM.pdf]

Description of Additional Supplementary Files

File Name: Supplementary Data 1
Description: Predicted TrpL leader peptide sequences from candidate species identified by Merino, et al. (2008).

File Name: Supplementary Data 2
Description: Complete list of oligonucleotide primers used in this study.

File Name: Supplementary Data 3
Description: Nucleotide sequences generated from 5' pRACE analysis.

File Name: Supplementary Data 4
Description: Nucleotide BLAST results for 5' pRACE sequencing analysis.

File Name: Supplementary Data 5
Description: File containing all amino acid sequences of CTL0325 (YtgCR) and CTL0174 used for multiple sequence alignments.

File Name: Supplementary Data 6
Description: Complete multiple sequence alignments for CTL0174.

File Name: Supplementary Data 7
Description: Complete multiple sequence alignments for CTL0325 (YtgCR).

File Name: Supplementary Data 8
Description: Summary of PASIFIC analysis results.

File Name: Supplementary Data 9
Description: Nucleotide sequences used for PASIFIC analysis.

File Name: Supplementary Software 1
Description: File containing the source code for all figures and statistics generated in R Studio using ggplot2 and ggpubr packages.
