# Supplementary material for: The iron-dependent repressor YtgR is a tryptophan-dependent attenuator of the trpRBA operon in Chlamydia trachomatis
Source: Nat Commun. 2020 Dec 22;11:6430. doi: 10.1038/s41467-020-20181-5 (PMC7755916; doi:10.1038/s41467-020-20181-5)
Supplement: Supplementary file 14 — Reporting Summary [file 41467_2020_20181_MOESM14_ESM.pdf]

nature research

Corresponding author(s): Rey Carabeo

Last updated by author(s): Oct 28, 2020

Reporting Summary

Nature Research wishes to improve the reproducibility of the work that we publish. This form provides structure for consistency and transparency in reporting. For further information on Nature Research policies, see our Editorial Policies and the Editorial Policy Checklist.

Statistics

For all statistical analyses, confirm that the following items are present in the figure legend, table legend, main text, or Methods section.

n/a	Confirmed	
☐	☒	The exact sample size (*n*) for each experimental group/condition, given as a discrete number and unit of measurement
☐	☒	A statement on whether measurements were taken from distinct samples or whether the same sample was measured repeatedly
☐	☒	The statistical test(s) used AND whether they are one- or two-sided<br>*Only common tests should be described solely by name; describe more complex techniques in the Methods section.*
☒	☐	A description of all covariates tested
☐	☒	A description of any assumptions or corrections, such as tests of normality and adjustment for multiple comparisons
☐	☒	A full description of the statistical parameters including central tendency (e.g. means) or other basic estimates (e.g. regression coefficient) AND variation (e.g. standard deviation) or associated estimates of uncertainty (e.g. confidence intervals)
☐	☒	For null hypothesis testing, the test statistic (e.g. *F*, *t*, *r*) with confidence intervals, effect sizes, degrees of freedom and *P* value noted<br>*Give P values as exact values whenever suitable.*
☒	☐	For Bayesian analysis, information on the choice of priors and Markov chain Monte Carlo settings
☒	☐	For hierarchical and complex designs, identification of the appropriate level for tests and full reporting of outcomes
☒	☐	Estimates of effect sizes (e.g. Cohen's *d*, Pearson's *r*), indicating how they were calculated

Our web collection on statistics for biologists contains articles on many of the points above.

Software and code

Policy information about availability of computer code

Data collection	Images were acquired on Nikon Elements AR version 5.20.02 and Zeiss Zen version 2.3.
Data analysis	Image data were analyzed in Fiji ImageJ (version 2.0.0-rc-69/1.52p, build 269a0ad53f) and RT-qPCR and other non-image data were analyzed in R studio (version 1.2.1335) using the ggplot2 package (version 3.1.0) and the ggpubr package (version 0.2.3). Sequence analysis was performed in Mega7 (version 7180411) and Jalview (version 2.11.0).

For manuscripts utilizing custom algorithms or software that are central to the research but not yet described in published literature, software must be made available to editors and reviewers. We strongly encourage code deposition in a community repository (e.g. GitHub). See the Nature Research guidelines for submitting code & software for further information.

Data

Policy information about availability of data

All manuscripts must include a data availability statement. This statement should provide the following information, where applicable:

- Accession codes, unique identifiers, or web links for publicly available datasets
- A list of figures that have associated raw data
- A description of any restrictions on data availability

Any data that support the findings of this study beyond what is included in the Supplementary Information are available from the corresponding author upon request. Source data are provided with this paper in Supplementary Data 4. Requests for unique biological materials such as plasmids or transformants should be directed to the corresponding author. All genome sequence information was obtained from the National Center for Biotechnology Information (NCBI) Genome database (https://www.ncbi.nlm.nih.gov) and accession numbers to relevant genomes are referenced in the Methods where appropriate.

Field-specific reporting

Please select the one below that is the best fit for your research. If you are not sure, read the appropriate sections before making your selection.

- [x] Life sciences
- [ ] Behavioural & social sciences
- [ ] Ecological, evolutionary & environmental sciences

For a reference copy of the document with all sections, see nature.com/documents/nr-reporting-summary-flat.pdf

Life sciences study design

All studies must disclose on these points even when the disclosure is negative.

Sample size	Sample size for RT-qPCR experiments and immunoblot experiments was determined to be at least three (n=3) to provide sufficient data for the assumption of normality under the Central Limit Theorem. Both the t-test and Tukey's post-hoc test assume an underlying normally distributed population. For quantification of immunofluorescent signal, sample size was determined using a Power analysis based on the means derived from the first replicate collected for each experimental condition.
Data exclusions	No data were excluded for reasons other than random sampling.
Replication	All experiments were independently replicated at least three times. All replication attempts were successful.
Randomization	Consideration of covariates was not warranted under the given experimental conditions. Randomization of groups was therefore not necessary. All samples were treated side-by-side under the same conditions and compared to relevant controls.
Blinding	For random sampling of datasets, experimentally determined values were hidden and only sample ID was visible to randomly select values for statistical testing.

Reporting for specific materials, systems and methods

We require information from authors about some types of materials, experimental systems and methods used in many studies. Here, indicate whether each material, system or method listed is relevant to your study. If you are not sure if a list item applies to your research, read the appropriate section before selecting a response.

Materials & experimental systems

n/a	Involved in the study	
[ ]	[x]	Antibodies
[ ]	[x]	Eukaryotic cell lines
[x]	[ ]	Palaeontology and archaeology
[x]	[ ]	Animals and other organisms
[x]	[ ]	Human research participants
[x]	[ ]	Clinical data
[x]	[ ]	Dual use research of concern

Methods

n/a	Involved in the study	
[x]	[ ]	ChIP-seq
[x]	[ ]	Flow cytometry
[x]	[ ]	MRI-based neuroimaging

Antibodies

Antibodies used	Antibodies: polyclonal rabbit anti-TrpR (Li International, Denver, CO, USA); polyclonal rabbit anti-YtgR (Li International, Denver, CO, USA); polyclonal mouse anti-cHsp60 (Invitrogen, MA3-023); Normal Rabbit IgG (Cell Signaling Technology, #2729); monoclonal mouse anti-FLAG (Cell Signaling Technology, #8146); monoclonal rabbit anti-FLAG (Cell Signaling Technology, #14793); monoclonal mouse anti-MOMP (clone L2I-45, generated by and a gift of Dr. Harlan Caldwell, NIH); rabbit anti-mouse IgG-HRP (Agilent Dako, P0260); goat anti-rabbit IgG-HRP (Agilent Dako, P0448); goat anti-rabbit IgG-Alexafluor-594 (Invitrogen, ThermoFisher, A11012); goat anti-mouse IgG-Alexafluor-488 (Invitrogen, ThermoFisher, A11001)
Validation	anti-TrpR was validated in-house by western blotting for specificity and ChIP-qPCR conditions were optimized for efficient immunoprecipiation of TrpR-bound DNA fragments<br>anti-YtgR was validated in-house for specificity by optimization of western blotting conditions<br>anti-cHsp60 has been validated in several publications linked on the manufacturer webpage for immunoblotting, immunocytochemistry, immunohistochemistry and immunofluorescence.<br>mouse anti-FLAG has been validated by the manufacturer for immunoblotting, immunoprecipitation, immunohistochemistry, immunfluorescence and flow cytometry.<br>rabbit anti-FLAG has been validated by the manufacturer for immunoblotting, immunoprecipitation, immunohistochemistry, immunfluorescence, flow cytometry and ChIP.<br>Normal Rabbit IgG has been validated by the manufacturer for immunoprecipitation and ChIP.<br>anti-MOMP antibody was validated in-house for specificity by immunoblotting and immunofluorescence and by Caldwell group.<br>Dako HRP-conjugated secondary antibodies have been validated by the manufacturer for limited cross-reactivity.

Alexafluor-conjugated secondary antibodies have been validated by the manufacturer for immunofluorescence, among several other applications.

Eukaryotic cell lines

Policy information about cell lines

Cell line source(s)	HeLa cervical epithelial adenocarcinoma cells were originally acquired from ATCC<br>McCoy B mouse fibroblast cells were originally acquired from Dr. Harlan Caldwell (NIH) who acquired them from ATCC
Authentication	HeLa cells were originally authenticated by ATCC via STR profiling and isoenzyme analysis per ATCC specifications. Authentication of McCoy B cells is not specified on ATCC.
Mycoplasma contamination	To our knowledge, all cell lines used in this study were mycoplasma free as determined by routine PCR sampling for contamination.
Commonly misidentified lines (See ICLAC register)	No commonly misidentified cells were used in this study.
